# Insulinoma presenting as hypoglycemia during lactose tolerance testing: a case report

**DOI:** 10.1186/s13256-020-02419-4

**Published:** 2020-06-30

**Authors:** Vicki Munro, Laura M. McDonell, Valerie Keough, Ferhan S. Siddiqi

**Affiliations:** 1 Division of Endocrinology and Metabolism, Department of Medicine, Dalhousie University, Halifax, NS Canada; 2 Department of Pathology, Dalhousie University, Halifax, NS Canada; 3 Department of Diagnostic Radiology, Dalhousie University, Halifax, NS Canada; 4 Queen Elizabeth II Health Sciences Centre, 043-7 North Victoria Building, 1276 South Park Street, Halifax, NS B3H 2Y9 Canada

**Keywords:** Insulinoma, Pancreatic neuroendocrine tumors, Hypoglycemia, Lactose tolerance testing, Case report

## Abstract

**Background:**

Insulinoma is a rare functioning pancreatic endocrine tumor, typically presenting as a sporadic solitary lesion causing hypoglycemia. While these tumors can lead to marked autonomic and neuroglycopenic symptoms, the diagnosis is often delayed.

**Case presentation:**

We present a case of a 60-year-old Caucasian man presenting with a 1-year history of progressive episodic confusion and an unexpected finding of symptomatic hypoglycemia during a lactose tolerance test. Further inquiry revealed an 8-year history of more subtle episodic neuroglycopenic symptoms preceding his presentation. After additional biochemical testing suggested a diagnosis of insulinoma, abdominal imaging was performed and revealed a 1.2-cm tumor in the tail of the pancreas. Following laparoscopic resection of the tumor, the patient had complete resolution of his symptoms and maintained normal glucose levels.

**Conclusions:**

The clinical presentation of functioning pancreatic neuroendocrine tumors can be subtle and nonspecific. As such, clinicians should remain vigilant for insulinoma when symptomatic hypoglycemia is present. To our knowledge, this is the first report of an insulinoma found after hypoglycemia was detected during lactose tolerance testing.

## Background

Insulinomas are the most common type of functioning pancreatic neuroendocrine tumors. Their incidence is estimated at about 4 cases per 1 million person-years [1]. The majority are benign, solitary, and follow an indolent course [2]. Patients with insulinomas most commonly present with fasting hypoglycemia, typically associated with neuroglycopenic symptoms such as confusion, behavior changes, difficulty with concentration, dizziness, blurred vision, paresthesias, and seizures. The presence of the Whipple triad, which consists of low blood glucose less than 2.8 mmol/L (50 mg/dl), hypoglycemic symptoms, and relief of symptoms following glucose ingestion, is a hallmark of this condition. However, the diagnosis is often delayed by years because insulinomas are infrequently encountered and generally present with nonspecific symptoms that may sometimes mimic a neurological disorder. In this report, we present a case of a patient with many years of nonspecific neurologic and abdominal symptoms who experienced marked hypoglycemia during lactose tolerance testing and was ultimately diagnosed with an insulinoma.

## Case presentation

A 60-year-old Caucasian man presented to our hospital with a history of episodic confusion over the course of 1 year. Specifically, he described intermittent disorientation and difficulty concentrating at work with brief amnesic spells. At home, he would episodically speak to his wife in an incoherent manner. In retrospect, his symptoms went back 7 to 8 years, although these initial episodes were more often preceded by hunger, blurred vision, and occasionally paresthesias. Further history taking revealed that all episodes happened after a prolonged fast or in the absence of food for at least 4 to 5 hours. The symptoms would predictably and almost immediately resolve with eating food or drinking juice. He had no associated tremor or diaphoresis. He did not report any history of seizures or syncope. Notably, he had experienced a weight gain of almost 10 pounds over the last year. He also complained of persistent abdominal discomfort and bloating.

His past medical history included lymphedema, gout, and intraocular lens replacement. He was not receiving any prescription medications. He was taking supplements of vitamin D, fish oil, vitamin B complex, and probiotics. His family history was significant for pancreatic cancer in his mother.

Prior to his referral to endocrinology, he was also being investigated for vague abdominal symptoms. During a lactose tolerance test, he began having a symptomatic episode and was found to have a very low serum blood glucose of 1.2 mmol/L. The lactose tolerance test checks for glucose levels rising as lactose breaks down. After being provided a glucose meter to check his blood sugar, he recorded many days of consistently low fasting morning capillary glucose readings ranging between 2.2 and 3.2 mmol/L. He noted that his cognition would improve after oral glucose ingestion and normalization of his glucose values to between 5 and 7 mmol/L. He was referred to endocrinology for further assessment.

### Investigations

Investigations performed after an overnight fast revealed a morning serum glucose level of 1.9 mmol/L, insulin level of 141 pmol/L (normal range, less than 20.8 pmol/L [3 mU/L] during hypoglycemia), and C-peptide level of 1106 pmol/L (normal reference range, 260–1730 pmol/L). Results of laboratory tests for his serum ketones and anti-insulin antibodies were negative. A sulfonylurea drug screen was not performed, because this testing is not available at our local laboratory. However, he denied exogenous use of oral hypoglycemic agents or insulin. He had no history of liver disease or alcohol abuse. His liver transaminase levels were all within normal range. His lipase level was minimally elevated at 73 U/L (reference range, 13–60). Results of all other investigations were within normal limits, including serum creatinine (estimated glomerular filtration rate > 60 ml/minute), thyroid-stimulating hormone, morning cortisol, total calcium, albumin, and complete blood count. His hemoglobin A1c was 4.9%.

**Fig. 1:**
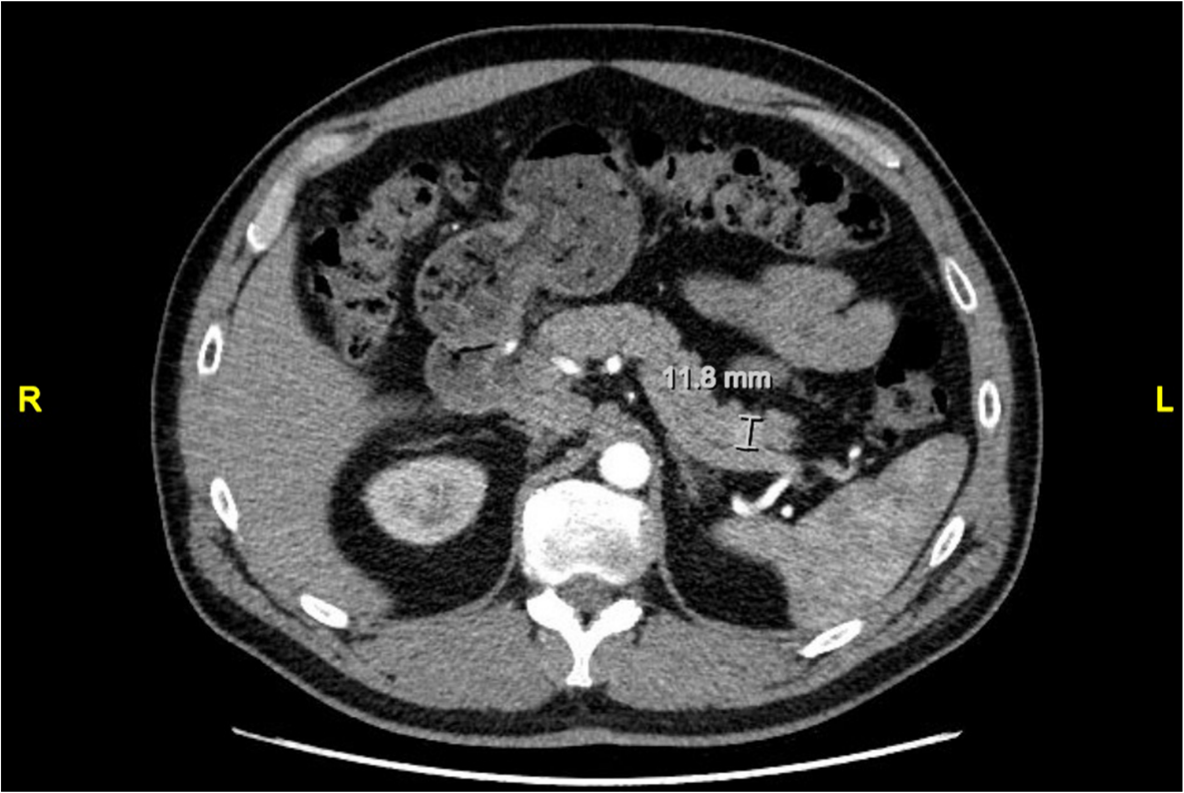
Computed tomographic scan of the abdomen showing a 1.2-cm tumor in the tail of the pancreas

An initial computed tomographic (CT) scan of the abdomen reported no significant abdominal or pancreatic pathology. However, using a pancreatic enhancement protocol, we identified a 1.3 × 1.2-cm mass lesion in the tail of the pancreas (Fig. 1). Magnetic resonance imaging (MRI) of his abdomen confirmed a similar-sized area of mild enhancement in the arterial phase, which was better visualized on subtraction sequence imaging. He had no evidence of metastasis. An octreotide scan was done but failed to show enhanced uptake in the area of the tail of the pancreas. He was subsequently referred to surgery and underwent successful laparoscopic distal pancreatectomy.

**Fig. 2:**
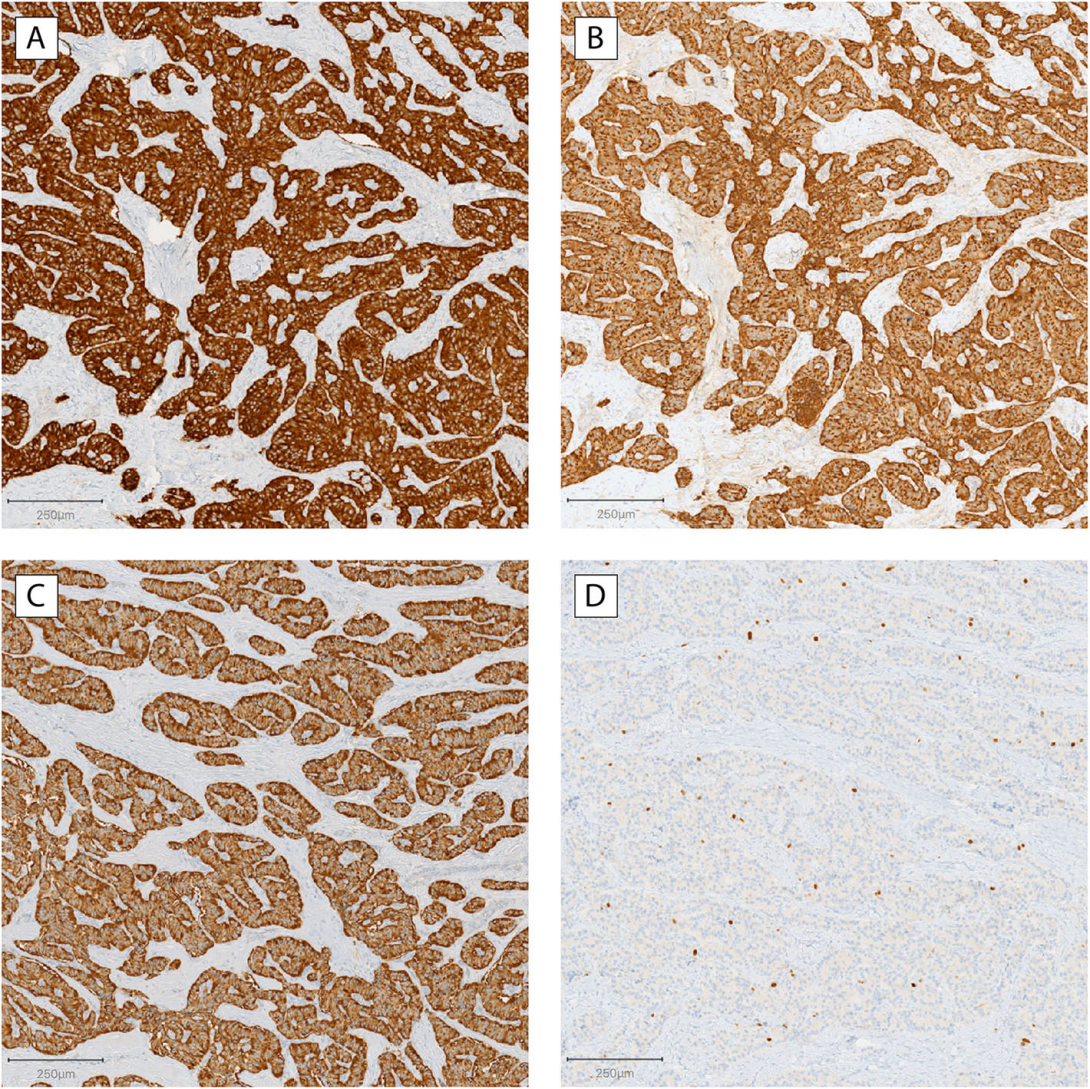
Immunohistochemistry of resected tumor showing pancreatic cells staining for (**a**) synaptophysin, (**b**) chromogranin A, (**c**) insulin, and (**d**) Ki-67. Neoplastic cells stained strongly positive for synaptophysin, chromogranin A, and insulin. The Ki-67 proliferative index was 3%

Sections of the resected tumor, which measured 1.2 cm in greatest dimension, showed a nested and trabecular proliferation of neoplastic cells separated by sclerotic stroma. Immunohistochemistry revealed that the neoplastic cells strongly expressed synaptophysin, chromogranin A, and insulin, confirming neuroendocrine differentiation and supporting the clinical diagnosis of insulinoma (Fig. 2a–c). The mitotic rate and Ki-67 proliferation index were 2 per 2 mm^2^ and 3%, respectively, in keeping with a well-differentiated (grade 2) pancreatic neuroendocrine tumor (Fig. 2d). The tumor was completely excised, and neither lymphovascular invasion nor lymph node involvement was identified.

### Outcome and follow-up

Surgical resection resulted in normalization of the patient’s glycemic values, resolution of episodic neuroglycopenic symptoms, and, interestingly, significant improvement of lymphedema. He continued to do well 5 years later with no evidence of recurrence, but he described some mild, persistent memory and cognitive deficits, which may be related to his previous hypoglycemic episodes. Given the association between insulinoma and multiple endocrine neoplasia type 1 (MEN1), he underwent genetic testing, but no known mutations were identified in *MEN1*.

## Discussion

Insulinomas are a form of well-differentiated functional pancreatic neuroendocrine tumor that most often present with hypoglycemia. Because patients may go many years before seeking medical attention, they do not always present with the classical sympathoadrenergic symptoms associated with hypoglycemia. Instead, they may present with an undiagnosed seizure disorder, unintended weight gain, or nonspecific symptoms such as confusion or abdominal pain [3–5]. Although the median delay between symptom onset and diagnosis is approximately 18 to 35 months [1, 5], some individuals are not diagnosed for decades. In this case, our patient’s initial subtle symptoms, which manifested 8 years prior to presentation, could likely correspond to the developing insulin-secreting tumor. It is then possible that his initial clinical symptoms of hunger and paresthesias were eventually replaced by progressive hypoglycemic unawareness due to the frequent and sustained episodes of hypoglycemia.

The biochemical diagnosis of an insulinoma requires inappropriate insulin secretion in the presence of unequivocal hypoglycemia. The most commonly performed testing involves the measurement of plasma insulin and C-peptide levels during hypoglycemia (serum glucose less than 2.8 mmol/L), typically in the setting of a supervised inpatient 72-hour fast. The absence of ketones (for example, β-hydroxybutyrate) confirms an insulin-replete state as opposed to other causes of non-insulin-mediated hypoglycemia. Insulin antibodies can also be measured at any time to investigate autoimmune-related causes. Although the inpatient 72-hour fast has traditionally been part of the insulinoma workup, increasing bed occupancy pressures at our center, among other factors, made such formal evaluation less feasible. There is a shifting trend toward outpatient evaluation of patients with symptomatic hypoglycemia using a prolonged overnight fast with repeat morning glucose measurements and formal testing for insulin and C-peptide levels once the glucose level is low [6]. The use of continuous glucose monitoring has also received recent attention for its potential role in the diagnosis and management of insulinoma, including its utility in revealing hypoglycemia unawareness [7], during pregnancy [8], and for perioperative glucose monitoring during surgery [9]. In our case, the patient had successful outpatient biochemical testing that obviated the need for an inpatient stay or continuous glucose monitoring.

Although insulinomas classically cause fasting hypoglycemia, they are also known to lead to postprandial hypoglycemia. In one review, 21% of individuals with insulinomas experienced both fasting and postprandial hypoglycemia, whereas only 6% experienced postprandial hypoglycemia exclusively [6]. Those with postprandial symptoms can be tested with random or mixed meal testing rather than the inpatient 72-hour fast. In our case, the patient primarily described episodes of fasting hypoglycemia, but, interestingly, it was possible that postprandial hypoglycemia during a lactose tolerance test led to his referral to endocrinology.

Following biochemical diagnosis, localization of the tumor is pursued by noninvasive abdominal imaging. The sensitivity of transabdominal ultrasound in the localization of insulinomas is rather poor (ranging from 9% to 64%) [10]. A contrast-enhanced CT scan is considered the imaging modality of choice. Using a thin-section multidetector CT technique can increase the sensitivity to almost 95% [11]. MRI also provides high sensitivity in detecting insulinomas because they generally have low signal intensity on T1-weighted imaging and high signal intensity on T2-weighted imaging [12] and tend to show relatively avid contrast enhancement on pancreatic parenchymal phase sequences. The use of ^111^In-octreotide scintigraphy is relatively common, but it has limited utility because many tumors lack the expression of somatostatin subtype 2 receptors required for detection. Finally, there may be an emerging role for ^68^Ga-DOTATATE positron emission tomography for accurate localization of insulinomas. A small retrospective study showed that ^68^Ga-DOTATATE successfully identified an insulinoma in nine of ten individuals (90%), including one patient in whom the results of all other noninvasive imaging modalities were negative [13]. As novel cell surface targets such as glucagon-like peptide-1 (GLP-1) receptors become identified, additional targets for radioisotope-labeled scintigraphy may be developed and integrated for improved detection of pancreatic neuroendocrine tumors [14].

Preoperative localization of insulinomas has become increasingly important for surgical planning and management. As such, the use of invasive imaging techniques, including endoscopic ultrasound (EUS) and selective arterial calcium stimulation testing, is becoming more frequent. The sensitivities of EUS and arterial calcium stimulation are very high [6], but these procedures are operator-dependent and may not be available at every center. In addition, EUS may not detect insulinomas located in the distal pancreas [15]. This highlights the important practical limitations of invasive testing, which must be weighed against the value of preoperative tumor localization that other noninvasive imaging may have missed.

Surgical resection of the insulinoma is the treatment of choice. The majority of patients with insulinomas, in particular those with smaller tumors (< 2 cm) and low Ki-67 proliferation indexes, have excellent long-term survival following surgical excision of the tumor [1]. Minimally invasive surgical techniques, including laparoscopic surgery, provide similar patient outcomes and shorter hospital stays compared with open pancreatic surgery [16]. Persistence of hypoglycemic symptoms should prompt investigation for additional insulinomas or, rarely, metastases. The risk of recurrence is more common in patients with *MEN1*. Insulinomas arising from *MEN1* tend to occur at a younger age, often before 40 years of age, and may be multifocal at diagnosis [2]. In these cases, thorough presurgical planning may be required to identify all lesions, and surgical resection may be more extensive. Our patient had a negative targeted genetic test result for *MEN1* mutations.

## Conclusion

In summary, our patient presented with a protracted history of undiagnosed neurologic symptoms secondary to recurrent hypoglycemia induced by a solitary pancreatic insulinoma. Remarkably, he was diagnosed with hypoglycemia for the first time during a lactose tolerance test for nonspecific abdominal symptoms, which ultimately led to outpatient biochemical workup for his unexpected hypoglycemia. Elevated outpatient insulin and C-peptide levels associated with fasting hypoglycemia confirmed the clinical diagnosis of insulinoma. A 1.2-cm tumor was identified in the tail of the pancreas by contrast-enhanced CT and MRI, and the patient underwent successful laparoscopic resection. He has demonstrated an excellent long-term outcome with no evidence of recurrence 5 years later. To our knowledge, this is the first reported case of a lactose tolerance test leading to the diagnosis of an insulinoma, highlighting the importance of broad investigations and astute clinical judgment in patients with nonspecific presentations.

## Data Availability

Not applicable.
